# 4-(2,4-Dichloro­phen­yl)-5,5-dimethyl-2-(3-silatranyl­propyl­mino)-1,3,2-dioxa­phospho­rinane 2-oxide

**DOI:** 10.1107/S1600536811044928

**Published:** 2011-11-12

**Authors:** Zhe-Rong Liu, Xiao-Jing Tan, De-Jian Wang, Yan Wang

**Affiliations:** a School of Chemical and Environmental Engineering, Hubei University for Nationalities, Enshi, Hubei 445000, Peoples’ Republic of China

## Abstract

In the title compound, C_20_H_31_Cl_2_N_2_O_6_PSi, the dioxaphospho­rinane ring adopts a *cis* conformation. The silatrane fragment forms a cage-like structure in which there exists an intra­molecular Si—N donor–acceptor bond. In the crystal, centrosymmetrically related mol­ecules are linked by pairs of N—H⋯O hydrogen bonds into inversion dimers, generating rings with graph-set motif *R*_2_^2^(8). The dimers are further connected into ribbons parallel to the *a* axis by inter­molecular C—H⋯O hydrogen bonds.

## Related literature

For the biological activity of 1,3,2-dioxaphospho­rinane compounds, see: Shi *et al.* (2006 ▶); Sun *et al.* (2006 ▶) and of γ-amino­propyl­silatrane, see: Puri *et al.* (2011 ▶). For the synthesis of the title compound, see: Wan *et al.* (2005 ▶).
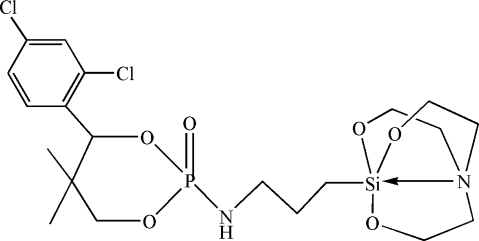


## Experimental

### 

#### Crystal data

C_20_H_31_Cl_2_N_2_O_6_PSi
*M* *_r_* = 525.43
Triclinic, 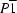

*a* = 10.7738 (12) Å
*b* = 10.9320 (13) Å
*c* = 11.2807 (13) Å
α = 111.135 (2)°
β = 95.926 (2)°
γ = 90.424 (2)°
*V* = 1231.2 (2) Å^3^
*Z* = 2
Mo *K*α radiation
μ = 0.42 mm^−1^
*T* = 298 K
0.20 × 0.15 × 0.08 mm

#### Data collection

Bruker SMART CCD area-detector diffractometer
6745 measured reflections
4720 independent reflections
2997 reflections with *I* > 2σ(*I*)
*R*_int_ = 0.033

#### Refinement

*R*[*F* ^2^ > 2σ(*F* ^2^)] = 0.065
*wR*(*F* ^2^) = 0.194
*S* = 1.00
4720 reflections
291 parameters
H-atom parameters constrained
Δρ_max_ = 0.36 e Å^−3^
Δρ_min_ = −0.35 e Å^−3^

### 

Data collection: *SMART* (Bruker, 2001 ▶); cell refinement: *SAINT* (Bruker, 2001 ▶); data reduction: *SAINT*; program(s) used to solve structure: *SHELXS97* (Sheldrick, 2008 ▶); program(s) used to refine structure: *SHELXL97* (Sheldrick, 2008 ▶); molecular graphics: *SHELXTL* (Sheldrick, 2008 ▶); software used to prepare material for publication: *SHELXTL*.

## Supplementary Material

Crystal structure: contains datablock(s) global, I. DOI: 10.1107/S1600536811044928/rz2656sup1.cif

Structure factors: contains datablock(s) I. DOI: 10.1107/S1600536811044928/rz2656Isup2.hkl

Additional supplementary materials:  crystallographic information; 3D view; checkCIF report

## Figures and Tables

**Table 1:** Hydrogen-bond geometry (Å, °)

*D*—H⋯*A*	*D*—H	H⋯*A*	*D*⋯*A*	*D*—H⋯*A*
N1—H1⋯O3^i^	0.86	2.05	2.857 (5)	155
C6—H6⋯O6^ii^	0.93	2.45	3.332 (6)	158

Symmetry codes: (i) 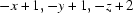; (ii) 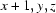.

**Fig. 1.**
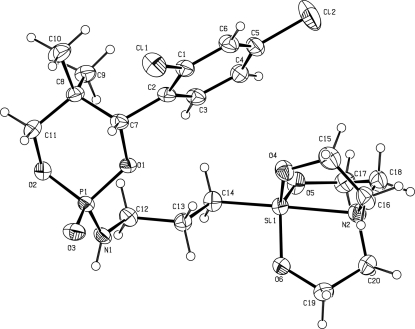
The molecular structure of the title compound with displacement ellipsoids drawn at the 50% probability level. H atoms are represented by circles of arbitrary size.

**Fig. 2.**
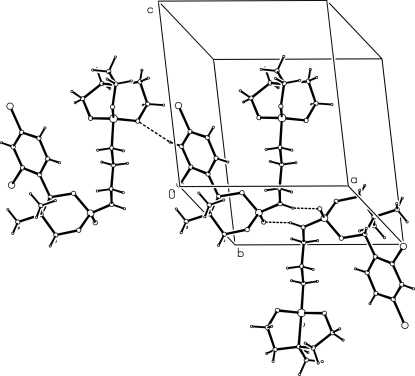
A partial packing diagram of ther title compound, showing the intermolecular hydrogen bonds (dashed lines).

## supplementary crystallographic information

### Comment

1,3,2-Dioxaphosphorinane compounds have attracted many chemists' interest owing to their stereochemistry and wide biological activities, such as fungicidal, insecticidal as well as herbicidal activities (Shi *et al.*, 2006; Sun *et al.*, 2006). γ-Aminopropylsilatrane has been found to have good biological activity (Puri *et al.*, 2011). In view of this and as a continuation of our research on the stereochemistry and biological properties of this class of compounds, we investigates 1,3,2-dioxaphosphinane derivatives containing γ-aminopropyl silatrane, including the title compound, (I), whose crystal structure is reported herein.

The crystal structure of (I) (Fig. 1) reveals that the cyclic dioxaphosphorinane ring in the molecule adopts a thermodynamically stable *cis* conformation, while the silatrane fragment forms a cage-like structure in which there exists an intramolecular Si←N donor-acceptor bond (Si1—N2 = 2.148 (4) Å), which is remarkably longer than an usual Si—N single bond (1.7–1.8 Å). In the crystal structure, molecules are linked by pairs of complementary N—H···O hydrogen bonds into centrosymmetric dimers, forming rings of graph-set motif *R*_2_^2^(8) (Table 1; Fig. 2). The dimers are further linked into ribbon parallel to the *a* axis by intermolecular C—H···O hydrogen bonds.

### Experimental

The title compound was prepared according to the procedure of Wan *et al.* (2005). Suitable crystals were obtained by vapor diffusion of tetrahydrofuran (THF) at room temperature (m.p. 522–523 K). Elemental analysis: calculated for C_20_H_31_Cl_2_N_2_O_6_PSi: C 45.72, H 5.95, N 5.33%; found: C 45.89, H 6.11, N 5.22%.

### Refinement

All H atoms were positioned geometrically and constrained to ride on their parent atoms, with C—H = 0.93–0.98 Å, N—H = 0.86Å, and with *U*_iso_(H) = 1.2*U*_eq_(C, N) or 1.5*U*_eq_(C) for methyl H atoms.

### Crystal data

**Table:**

C_20_H_31_Cl_2_N_2_O_6_PSi	*Z* = 2
*M**_r_* = 525.43	*F*(000) = 552
Triclinic, *P*1	*D*_x_ = 1.417 Mg m^−^^3^*D*_m_ = 1.417 Mg m^−^^3^*D*_m_ measured by not measured
Hall symbol: -P 1	Melting point = 522–523 K
*a* = 10.7738 (12) Å	Mo *K*α radiation, λ = 0.71073 Å
*b* = 10.9320 (13) Å	Cell parameters from 1174 reflections
*c* = 11.2807 (13) Å	θ = 2.2–19.3°
α = 111.135 (2)°	µ = 0.42 mm^−^^1^
β = 95.926 (2)°	*T* = 298 K
γ = 90.424 (2)°	Needle, colorless
*V* = 1231.2 (2) Å^3^	0.20 × 0.15 × 0.08 mm

### Data collection

**Table:**

Bruker SMART CCD area-detector diffractometer	2997 reflections with *I* > 2σ(*I*)
Radiation source: fine-focus sealed tube	*R*_int_ = 0.033
graphite	θ_max_ = 26.0°, θ_min_ = 1.9°
φ and ω scans	*h* = −13→6
6745 measured reflections	*k* = −13→13
4720 independent reflections	*l* = −13→13

### Refinement

**Table:**

Refinement on *F*^2^	Primary atom site location: structure-invariant direct methods
Least-squares matrix: full	Secondary atom site location: difference Fourier map
*R*[*F*^2^ > 2σ(*F*^2^)] = 0.065	Hydrogen site location: inferred from neighbouring sites
*wR*(*F*^2^) = 0.194	H-atom parameters constrained
*S* = 1.00	*w* = 1/[σ^2^(*F*_o_^2^) + (0.0969*P*)^2^] where *P* = (*F*_o_^2^ + 2*F*_c_^2^)/3
4720 reflections	(Δ/σ)_max_ < 0.001
291 parameters	Δρ_max_ = 0.36 e Å^−^^3^
0 restraints	Δρ_min_ = −0.35 e Å^−^^3^

### Special details

**Table:**

Geometry. All e.s.d.'s (except the e.s.d. in the dihedral angle between two l.s. planes) are estimated using the full covariance matrix. The cell e.s.d.'s are taken into account individually in the estimation of e.s.d.'s in distances, angles and torsion angles; correlations between e.s.d.'s in cell parameters are only used when they are defined by crystal symmetry. An approximate (isotropic) treatment of cell e.s.d.'s is used for estimating e.s.d.'s involving l.s. planes.
Refinement. Refinement of *F*^2^ against ALL reflections. The weighted *R*-factor *wR* and goodness of fit *S* are based on *F*^2^, conventional *R*-factors *R* are based on *F*, with *F* set to zero for negative *F*^2^. The threshold expression of *F*^2^ > σ(*F*^2^) is used only for calculating *R*-factors(gt) *etc*. and is not relevant to the choice of reflections for refinement. *R*-factors based on *F*^2^ are statistically about twice as large as those based on *F*, and *R*- factors based on ALL data will be even larger.

### Fractional atomic coordinates and isotropic or equivalent isotropic displacement parameters (Å^2^)

**Table:**

	*x*	*y*	*z*	*U*_iso_*/*U*_eq_	
C1	1.0122 (4)	0.6821 (4)	0.7954 (4)	0.0437 (11)	
C2	0.9035 (4)	0.7254 (4)	0.8460 (4)	0.0358 (9)	
C3	0.8260 (4)	0.7889 (4)	0.7813 (4)	0.0438 (11)	
H3	0.7504	0.8184	0.8114	0.053*	
C4	0.8579 (4)	0.8088 (4)	0.6755 (4)	0.0457 (11)	
H4	0.8037	0.8499	0.6337	0.055*	
C5	0.9678 (5)	0.7691 (5)	0.6316 (4)	0.0514 (12)	
C6	1.0482 (4)	0.7019 (5)	0.6884 (4)	0.0501 (12)	
H6	1.1228	0.6715	0.6563	0.060*	
C7	0.8618 (4)	0.7051 (4)	0.9614 (4)	0.0379 (10)	
H7	0.8979	0.6255	0.9680	0.045*	
C8	0.8945 (4)	0.8210 (4)	1.0912 (4)	0.0425 (10)	
C9	0.8522 (4)	0.9521 (4)	1.0853 (5)	0.0531 (12)	
H9A	0.7647	0.9443	1.0554	0.080*	
H9B	0.8986	0.9753	1.0277	0.080*	
H9C	0.8666	1.0190	1.1690	0.080*	
C10	1.0350 (4)	0.8302 (5)	1.1341 (5)	0.0645 (15)	
H10A	1.0800	0.8493	1.0731	0.097*	
H10B	1.0601	0.7481	1.1395	0.097*	
H10C	1.0530	0.8988	1.2164	0.097*	
C11	0.8290 (5)	0.7924 (5)	1.1928 (4)	0.0567 (13)	
H11A	0.8597	0.7124	1.2011	0.068*	
H11B	0.8499	0.8634	1.2745	0.068*	
C12	0.4695 (4)	0.7914 (4)	0.9460 (4)	0.0436 (10)	
H12A	0.5321	0.8633	0.9787	0.052*	
H12B	0.3910	0.8250	0.9763	0.052*	
C13	0.4549 (4)	0.7405 (4)	0.8012 (4)	0.0423 (10)	
H13A	0.3894	0.6714	0.7699	0.051*	
H13B	0.5320	0.7014	0.7720	0.051*	
C14	0.4239 (4)	0.8422 (4)	0.7415 (4)	0.0454 (11)	
H14A	0.4879	0.9126	0.7744	0.054*	
H14B	0.3453	0.8793	0.7680	0.054*	
C15	0.6068 (5)	0.6880 (5)	0.4263 (5)	0.0603 (13)	
H15A	0.6707	0.6263	0.4277	0.072*	
H15B	0.6443	0.7597	0.4088	0.072*	
C16	0.5014 (5)	0.6206 (5)	0.3235 (4)	0.0556 (13)	
H16A	0.5233	0.6140	0.2402	0.067*	
H16B	0.4830	0.5330	0.3218	0.067*	
C17	0.3320 (5)	0.9231 (4)	0.4212 (4)	0.0544 (13)	
H17A	0.3551	1.0093	0.4219	0.065*	
H17B	0.2420	0.9093	0.4015	0.065*	
C18	0.3951 (5)	0.8196 (5)	0.3220 (4)	0.0540 (12)	
H18A	0.3508	0.8010	0.2376	0.065*	
H18B	0.4805	0.8484	0.3219	0.065*	
C19	0.2604 (5)	0.5652 (5)	0.4171 (4)	0.0592 (13)	
H19A	0.1737	0.5394	0.4154	0.071*	
H19B	0.3090	0.4875	0.3989	0.071*	
C20	0.2724 (5)	0.6263 (5)	0.3168 (4)	0.0561 (13)	
H20A	0.2721	0.5589	0.2326	0.067*	
H20B	0.2042	0.6832	0.3148	0.067*	
Cl1	1.11497 (12)	0.59273 (13)	0.86003 (13)	0.0648 (4)	
Cl2	1.01035 (15)	0.8000 (2)	0.50014 (16)	0.1002 (7)	
N1	0.5068 (3)	0.6877 (3)	0.9955 (3)	0.0420 (9)	
H1	0.4481	0.6388	1.0041	0.050*	
N2	0.3930 (3)	0.7025 (3)	0.3565 (3)	0.0455 (9)	
O1	0.7265 (2)	0.6839 (3)	0.9344 (3)	0.0386 (7)	
O2	0.6939 (3)	0.7780 (3)	1.1629 (3)	0.0526 (8)	
O3	0.6623 (3)	0.5297 (3)	1.0444 (3)	0.0529 (8)	
O4	0.5583 (3)	0.7371 (3)	0.5471 (3)	0.0515 (8)	
O5	0.3694 (3)	0.9157 (3)	0.5428 (3)	0.0493 (8)	
O6	0.3041 (3)	0.6576 (3)	0.5381 (3)	0.0481 (8)	
P1	0.64665 (11)	0.65882 (11)	1.03461 (10)	0.0383 (3)	
Si1	0.41111 (11)	0.77709 (11)	0.56192 (11)	0.0373 (3)	

### Atomic displacement parameters (Å^2^)

**Table:**

	*U*^11^	*U*^22^	*U*^33^	*U*^12^	*U*^13^	*U*^23^
C1	0.036 (3)	0.049 (3)	0.051 (3)	−0.001 (2)	0.000 (2)	0.027 (2)
C2	0.028 (2)	0.036 (2)	0.048 (2)	0.0010 (18)	0.0038 (18)	0.0212 (19)
C3	0.033 (2)	0.050 (3)	0.055 (3)	0.000 (2)	0.001 (2)	0.028 (2)
C4	0.037 (3)	0.059 (3)	0.047 (3)	0.000 (2)	−0.001 (2)	0.027 (2)
C5	0.047 (3)	0.067 (3)	0.050 (3)	−0.003 (2)	0.005 (2)	0.033 (2)
C6	0.038 (3)	0.060 (3)	0.057 (3)	0.001 (2)	0.009 (2)	0.027 (2)
C7	0.034 (2)	0.038 (2)	0.047 (2)	−0.0011 (18)	0.0017 (19)	0.023 (2)
C8	0.036 (3)	0.047 (3)	0.045 (3)	−0.008 (2)	−0.0044 (19)	0.021 (2)
C9	0.053 (3)	0.044 (3)	0.058 (3)	−0.010 (2)	0.003 (2)	0.015 (2)
C10	0.049 (3)	0.070 (4)	0.067 (3)	−0.012 (3)	−0.016 (3)	0.021 (3)
C11	0.059 (3)	0.065 (3)	0.041 (3)	−0.019 (3)	−0.010 (2)	0.018 (2)
C12	0.045 (3)	0.043 (2)	0.046 (3)	0.007 (2)	0.011 (2)	0.018 (2)
C13	0.048 (3)	0.036 (2)	0.041 (2)	0.002 (2)	0.005 (2)	0.0121 (19)
C14	0.052 (3)	0.041 (2)	0.045 (3)	0.009 (2)	0.006 (2)	0.017 (2)
C15	0.051 (3)	0.067 (3)	0.066 (3)	0.018 (3)	0.022 (3)	0.024 (3)
C16	0.067 (4)	0.054 (3)	0.047 (3)	0.015 (3)	0.028 (2)	0.013 (2)
C17	0.062 (3)	0.047 (3)	0.060 (3)	0.002 (2)	−0.007 (2)	0.029 (2)
C18	0.066 (3)	0.053 (3)	0.046 (3)	0.001 (2)	0.005 (2)	0.023 (2)
C19	0.068 (4)	0.052 (3)	0.051 (3)	−0.016 (3)	0.007 (3)	0.012 (2)
C20	0.067 (4)	0.053 (3)	0.041 (3)	−0.009 (2)	0.003 (2)	0.009 (2)
Cl1	0.0502 (8)	0.0797 (9)	0.0828 (10)	0.0257 (7)	0.0131 (7)	0.0496 (8)
Cl2	0.0808 (12)	0.1756 (19)	0.0855 (11)	0.0259 (11)	0.0294 (9)	0.0912 (13)
N1	0.041 (2)	0.051 (2)	0.045 (2)	−0.0031 (17)	0.0070 (16)	0.0286 (18)
N2	0.054 (3)	0.041 (2)	0.043 (2)	0.0000 (18)	0.0076 (18)	0.0159 (17)
O1	0.0308 (16)	0.0466 (17)	0.0395 (16)	−0.0060 (13)	0.0009 (12)	0.0180 (13)
O2	0.052 (2)	0.066 (2)	0.0388 (17)	−0.0108 (16)	0.0013 (15)	0.0198 (16)
O3	0.0414 (19)	0.0554 (19)	0.072 (2)	−0.0042 (15)	−0.0008 (15)	0.0372 (17)
O4	0.0430 (19)	0.068 (2)	0.0493 (19)	0.0093 (16)	0.0092 (15)	0.0270 (16)
O5	0.068 (2)	0.0364 (16)	0.0422 (17)	0.0120 (15)	0.0025 (15)	0.0140 (14)
O6	0.054 (2)	0.0482 (18)	0.0408 (17)	−0.0099 (15)	0.0080 (14)	0.0145 (14)
P1	0.0384 (7)	0.0428 (7)	0.0382 (6)	−0.0051 (5)	0.0029 (5)	0.0204 (5)
Si1	0.0385 (7)	0.0367 (7)	0.0372 (6)	0.0038 (5)	0.0048 (5)	0.0140 (5)

### Geometric parameters (Å, °)

**Table:**

C1—C2	1.368 (6)	C14—Si1	1.879 (4)
C1—C6	1.392 (6)	C14—H14A	0.9700
C1—Cl1	1.754 (4)	C14—H14B	0.9700
C2—C3	1.400 (5)	C15—O4	1.429 (5)
C2—C7	1.508 (5)	C15—C16	1.509 (7)
C3—C4	1.365 (6)	C15—H15A	0.9700
C3—H3	0.9300	C15—H15B	0.9700
C4—C5	1.345 (6)	C16—N2	1.472 (6)
C4—H4	0.9300	C16—H16A	0.9700
C5—C6	1.390 (6)	C16—H16B	0.9700
C5—Cl2	1.739 (5)	C17—O5	1.420 (5)
C6—H6	0.9300	C17—C18	1.501 (7)
C7—O1	1.458 (5)	C17—H17A	0.9700
C7—C8	1.557 (6)	C17—H17B	0.9700
C7—H7	0.9800	C18—N2	1.467 (5)
C8—C11	1.527 (6)	C18—H18A	0.9700
C8—C9	1.529 (6)	C18—H18B	0.9700
C8—C10	1.532 (6)	C19—O6	1.405 (5)
C9—H9A	0.9600	C19—C20	1.522 (6)
C9—H9B	0.9600	C19—H19A	0.9700
C9—H9C	0.9600	C19—H19B	0.9700
C10—H10A	0.9600	C20—N2	1.475 (6)
C10—H10B	0.9600	C20—H20A	0.9700
C10—H10C	0.9600	C20—H20B	0.9700
C11—O2	1.454 (5)	N1—P1	1.592 (3)
C11—H11A	0.9700	N1—H1	0.8600
C11—H11B	0.9700	N2—Si1	2.148 (4)
C12—N1	1.474 (5)	O1—P1	1.585 (3)
C12—C13	1.514 (5)	O2—P1	1.590 (3)
C12—H12A	0.9700	O3—P1	1.465 (3)
C12—H12B	0.9700	O4—Si1	1.657 (3)
C13—C14	1.517 (5)	O5—Si1	1.665 (3)
C13—H13A	0.9700	O6—Si1	1.664 (3)
C13—H13B	0.9700		
			
C2—C1—C6	123.3 (4)	O4—C15—C16	108.8 (4)
C2—C1—Cl1	121.6 (3)	O4—C15—H15A	109.9
C6—C1—Cl1	115.1 (3)	C16—C15—H15A	109.9
C1—C2—C3	116.1 (4)	O4—C15—H15B	109.9
C1—C2—C7	124.6 (4)	C16—C15—H15B	109.9
C3—C2—C7	119.3 (4)	H15A—C15—H15B	108.3
C4—C3—C2	122.1 (4)	N2—C16—C15	106.1 (4)
C4—C3—H3	119.0	N2—C16—H16A	110.5
C2—C3—H3	119.0	C15—C16—H16A	110.5
C5—C4—C3	120.0 (4)	N2—C16—H16B	110.5
C5—C4—H4	120.0	C15—C16—H16B	110.5
C3—C4—H4	120.0	H16A—C16—H16B	108.7
C4—C5—C6	121.3 (4)	O5—C17—C18	108.9 (4)
C4—C5—Cl2	119.7 (4)	O5—C17—H17A	109.9
C6—C5—Cl2	118.9 (4)	C18—C17—H17A	109.9
C5—C6—C1	117.2 (4)	O5—C17—H17B	109.9
C5—C6—H6	121.4	C18—C17—H17B	109.9
C1—C6—H6	121.4	H17A—C17—H17B	108.3
O1—C7—C2	105.1 (3)	N2—C18—C17	106.2 (4)
O1—C7—C8	109.2 (3)	N2—C18—H18A	110.5
C2—C7—C8	115.6 (3)	C17—C18—H18A	110.5
O1—C7—H7	108.9	N2—C18—H18B	110.5
C2—C7—H7	108.9	C17—C18—H18B	110.5
C8—C7—H7	108.9	H18A—C18—H18B	108.7
C11—C8—C9	108.6 (4)	O6—C19—C20	108.9 (4)
C11—C8—C10	106.7 (4)	O6—C19—H19A	109.9
C9—C8—C10	110.2 (4)	C20—C19—H19A	109.9
C11—C8—C7	108.3 (3)	O6—C19—H19B	109.9
C9—C8—C7	112.3 (3)	C20—C19—H19B	109.9
C10—C8—C7	110.5 (4)	H19A—C19—H19B	108.3
C8—C9—H9A	109.5	N2—C20—C19	105.2 (4)
C8—C9—H9B	109.5	N2—C20—H20A	110.7
H9A—C9—H9B	109.5	C19—C20—H20A	110.7
C8—C9—H9C	109.5	N2—C20—H20B	110.7
H9A—C9—H9C	109.5	C19—C20—H20B	110.7
H9B—C9—H9C	109.5	H20A—C20—H20B	108.8
C8—C10—H10A	109.5	C12—N1—P1	125.4 (3)
C8—C10—H10B	109.5	C12—N1—H1	117.3
H10A—C10—H10B	109.5	P1—N1—H1	117.3
C8—C10—H10C	109.5	C18—N2—C16	114.4 (4)
H10A—C10—H10C	109.5	C18—N2—C20	113.7 (4)
H10B—C10—H10C	109.5	C16—N2—C20	113.3 (4)
O2—C11—C8	112.8 (3)	C18—N2—Si1	104.9 (3)
O2—C11—H11A	109.0	C16—N2—Si1	104.3 (3)
C8—C11—H11A	109.0	C20—N2—Si1	104.9 (3)
O2—C11—H11B	109.0	C7—O1—P1	120.6 (2)
C8—C11—H11B	109.0	C11—O2—P1	114.1 (3)
H11A—C11—H11B	107.8	C15—O4—Si1	122.9 (3)
N1—C12—C13	111.7 (3)	C17—O5—Si1	123.0 (3)
N1—C12—H12A	109.3	C19—O6—Si1	123.8 (3)
C13—C12—H12A	109.3	O3—P1—O1	113.75 (18)
N1—C12—H12B	109.3	O3—P1—O2	113.69 (17)
C13—C12—H12B	109.3	O1—P1—O2	102.17 (16)
H12A—C12—H12B	107.9	O3—P1—N1	114.75 (18)
C12—C13—C14	115.4 (3)	O1—P1—N1	105.51 (16)
C12—C13—H13A	108.4	O2—P1—N1	105.78 (18)
C14—C13—H13A	108.4	O4—Si1—O6	118.09 (17)
C12—C13—H13B	108.4	O4—Si1—O5	117.49 (17)
C14—C13—H13B	108.4	O6—Si1—O5	120.06 (17)
H13A—C13—H13B	107.5	O4—Si1—C14	97.52 (18)
C13—C14—Si1	114.4 (3)	O6—Si1—C14	96.54 (18)
C13—C14—H14A	108.7	O5—Si1—C14	96.87 (16)
Si1—C14—H14A	108.7	O4—Si1—N2	83.46 (15)
C13—C14—H14B	108.7	O6—Si1—N2	82.74 (14)
Si1—C14—H14B	108.7	O5—Si1—N2	82.90 (14)
H14A—C14—H14B	107.6	C14—Si1—N2	178.98 (19)
			
C6—C1—C2—C3	1.9 (6)	C19—C20—N2—Si1	−34.7 (4)
Cl1—C1—C2—C3	−176.4 (3)	C2—C7—O1—P1	−179.8 (2)
C6—C1—C2—C7	179.8 (4)	C8—C7—O1—P1	−55.2 (4)
Cl1—C1—C2—C7	1.5 (6)	C8—C11—O2—P1	62.2 (5)
C1—C2—C3—C4	−1.2 (6)	C16—C15—O4—Si1	−26.8 (5)
C7—C2—C3—C4	−179.2 (4)	C18—C17—O5—Si1	−28.5 (5)
C2—C3—C4—C5	−1.2 (7)	C20—C19—O6—Si1	−27.5 (5)
C3—C4—C5—C6	3.1 (7)	C7—O1—P1—O3	−71.7 (3)
C3—C4—C5—Cl2	−177.7 (4)	C7—O1—P1—O2	51.2 (3)
C4—C5—C6—C1	−2.4 (7)	C7—O1—P1—N1	161.7 (3)
Cl2—C5—C6—C1	178.4 (4)	C11—O2—P1—O3	71.4 (3)
C2—C1—C6—C5	−0.2 (7)	C11—O2—P1—O1	−51.6 (3)
Cl1—C1—C6—C5	178.2 (3)	C11—O2—P1—N1	−161.7 (3)
C1—C2—C7—O1	−143.9 (4)	C12—N1—P1—O3	−162.7 (3)
C3—C2—C7—O1	33.9 (5)	C12—N1—P1—O1	−36.6 (4)
C1—C2—C7—C8	95.6 (5)	C12—N1—P1—O2	71.2 (4)
C3—C2—C7—C8	−86.5 (5)	C15—O4—Si1—O6	82.5 (4)
O1—C7—C8—C11	53.4 (4)	C15—O4—Si1—O5	−74.1 (4)
C2—C7—C8—C11	171.6 (4)	C15—O4—Si1—C14	−175.9 (4)
O1—C7—C8—C9	−66.6 (4)	C15—O4—Si1—N2	4.3 (4)
C2—C7—C8—C9	51.6 (5)	C19—O6—Si1—O4	−73.6 (4)
O1—C7—C8—C10	169.9 (3)	C19—O6—Si1—O5	82.4 (4)
C2—C7—C8—C10	−71.8 (5)	C19—O6—Si1—C14	−175.7 (4)
C9—C8—C11—O2	62.5 (5)	C19—O6—Si1—N2	5.0 (3)
C10—C8—C11—O2	−178.7 (4)	C17—O5—Si1—O4	85.1 (4)
C7—C8—C11—O2	−59.7 (5)	C17—O5—Si1—O6	−71.1 (4)
N1—C12—C13—C14	−176.7 (4)	C17—O5—Si1—C14	−172.7 (4)
C12—C13—C14—Si1	178.2 (3)	C17—O5—Si1—N2	6.3 (4)
O4—C15—C16—N2	40.0 (5)	C13—C14—Si1—O4	−64.2 (4)
O5—C17—C18—N2	39.9 (5)	C13—C14—Si1—O6	55.4 (4)
O6—C19—C20—N2	40.0 (5)	C13—C14—Si1—O5	176.8 (3)
C13—C12—N1—P1	93.0 (4)	C18—N2—Si1—O4	−101.5 (3)
C17—C18—N2—C16	−147.6 (4)	C16—N2—Si1—O4	19.1 (3)
C17—C18—N2—C20	80.1 (4)	C20—N2—Si1—O4	138.4 (3)
C17—C18—N2—Si1	−33.9 (4)	C18—N2—Si1—O6	139.0 (3)
C15—C16—N2—C18	79.0 (5)	C16—N2—Si1—O6	−100.4 (3)
C15—C16—N2—C20	−148.5 (4)	C20—N2—Si1—O6	19.0 (3)
C15—C16—N2—Si1	−35.1 (4)	C18—N2—Si1—O5	17.4 (3)
C19—C20—N2—C18	−148.7 (4)	C16—N2—Si1—O5	138.0 (3)
C19—C20—N2—C16	78.4 (5)	C20—N2—Si1—O5	−102.7 (3)

### Hydrogen-bond geometry (Å, °)

**Table:**

*D*—H···*A*	*D*—H	H···*A*	*D*···*A*	*D*—H···*A*
N1—H1···O3^i^	0.86	2.05	2.857 (5)	155.
C6—H6···O6^ii^	0.93	2.45	3.332 (6)	158.

Symmetry codes: (i) −*x*+1, −*y*+1, −*z*+2; (ii) *x*+1, *y*, *z*.
